# Influence of Plasma Treatment on Surface Characteristics of Aluminum Alloy Sheets and Bonding Performance of Glass Fiber-Reinforced Thermoplastic/Al Composites

**DOI:** 10.3390/ma16093317

**Published:** 2023-04-23

**Authors:** Du-Cheng Tsai, Zue-Chin Chang, Erh-Chiang Chen, Yen-Lin Huang, Yun-Chen Jiang, Fuh-Sheng Shieu

**Affiliations:** 1 Department of Materials Science and Engineering, National Chung Hsing University, Taichung 402202, Taiwan; 2 Department of Mechanical Engineering, National Chin-Yi University of Technology, Taichung 411030, Taiwan; 3 Metal Industries Research and Development Centre, Kaohsiung 811225, Taiwan

**Keywords:** plasma treatment, contact angle, bonding strength, glass fiber-reinforced thermoplastic, surface wettability

## Abstract

This study focuses on modifying the surface of an AA 5052-H32 aluminum alloy using plasma treatment. Discharge power, exposure time, and working gas were adjusted as process parameters to improve the adhesion between the aluminum alloy and glass fiber-reinforced thermoplastic (GFRTP) polycarbonate composite. The surface composition and morphology of the aluminum alloy sheet were analyzed by X-ray photoelectron spectroscopy and scanning electron microscopy, and surface roughness and wettability were evaluated using a surface roughness-measuring instrument and contact angle goniometry, respectively. The bonding performance of GFRTP/aluminum alloy was also assessed. The surface of the aluminum alloy was subjected to chemical treatment prior to plasma treatment. The results revealed that nitrogen plasma treatment led to a substantial increase (25%) in bonding strength due to the synergistic effect of rough surface mechanical bonding and chemical bonding through functional groups between the aluminum alloy and GFRTP. However, the improvement in surface wettability by plasma treatment is time dependent and may gradually diminish over time due to the re-adsorption of hydrocarbon contamination from the surrounding air.

## 1. Introduction

Fiber metal laminates (FMLs) are a class of composite materials that offer a competitive alternative to traditional metals in various industrial applications. These materials exhibit exceptional mechanical properties and can be lightweight, making them highly attractive for use in weight-sensitive applications [1,2,3]. Among various FMLs, those composed of alternating layers of thin aluminum alloy and glass fiber-reinforced thermoplastic (GFRTP) have shown great potential in the automotive industry, rail transportation industry, construction industry, and aerospace industry, where high-performance and low-cost composite materials are in high demand. In comparison with traditional thermoset-based FMLs, thermoplastic-based FMLs offer several advantages. One of the main benefits of thermoplastic materials is their efficient production, which is highly attractive for industrial applications. Moreover, thermoplastic-based FMLs are highly recyclable, making them an ideal choice for sustainable applications. The thermal formability of thermoplastic materials further enhances their versatility, enabling them to be molded into various shapes to meet the diverse requirements of different applications [4,5].

FMLs require high interfacial adhesion between metal and composite layers to ensure a strong and durable joint that resists interlayer cracking and environmental stress. Various surface treatment methods are available to enhance the adhesion at the metal–composite interface of FMLs. Mechanical methods, such as grit blasting and mechanical abrasion, can increase surface roughness and create a peak-and-valley surface structure, leading to improved initial adhesion and bonding strength [6,7]. Mohamad et al. [8] explored the impact of aluminum surface roughness on the interfacial strength between metal and composites in FMLs, concluding that heightened surface roughness enhances bonding strength. Additionally, they observed that a high-roughness surface contributes to strong adhesion strength at the Al/GFRTP interface. Yao et al. [9] examined the influences of abrasion and grit blasting as two mechanical treatments on the metal–composite interface in FMLs. The findings indicate that grit blasting is a highly effective method to remarkably enhance interfacial fracture toughness in steel/GFRTP laminates. Sun et al. [10] revealed that sandblasting produces inferior results compared with micro-arc oxidation and laser ablation for adhesion at the Al/GFRTP interface. Chemical treatments can etch the metal surface, which has the dual benefit of removing the brittle oxide layer while generating a uniformly micro-roughened oxide layer, increasing the physicochemical activity for bonding substances and improving the adhesion at the metal–composite interface [11,12]. Prolongo et al. [13] reported that the surface properties of the 2024 aluminum alloy can be modified through P2 etching (a phosphate-fluoride process) to create a porous structure, resulting in high adhesion for epoxy/aluminum in FMLs. Aghamohammadi et al. [14] found that alkaline etching leads to the formation of a surface that is rough and flake-like, but this treatment does not contribute to improved adhesive bonding. Canche et al. [15] examined the efficacy of sanding, degreasing, and NaOH treatment at enhancing interfacial adhesion in thermoplastic-based FMLs. Their results demonstrated that the highest adhesion was attained following NaOH treatment, which resulted in the formation of surfaces that were cleaner, rougher, and more wettable. Electrochemical treatments, such as chromic acid anodizing, can also enhance adhesion by forming a porous, thin oxide layer with an increased surface area and polar groups on the metal surface. Huaguan [16] and Santos et al. [17] reported that the anodization of metal surfaces can generate uniformly porous pits and nanoscale porous oxide layers, providing excellent adhesion at the metal–composite interface. Moreover, combining anodizing with sandblasting, etching, and annealing on titanium surfaces can lead to the formation of a surface with hierarchical structures ranging from the nanoscale to the macroscale. [18].

Plasma surface technology has become a popular method for the surface modification of composites, metals, wood, carbon fibers, and implants owing to its superior efficiency and versatility [19,20,21,22]. The process combines plasma physics, plasma chemistry, and gas–solid interface reaction and can be completed quickly without affecting the properties of the materials. Several studies have shown that plasma surface treatment is effective at improving adhesive bond strength, surface energy, and surface roughness. Despite the success of plasma surface treatment in other applications, the potential of plasma treatment in FMLs remains largely unexplored, and the relevant parameters require further optimization to achieve the desired improvement in interfacial adhesion. Mui et al. [23] found that the superior adhesion between the paint coating and the Al alloy surface resulted from surface cleaning and activation rather than mechanical anchorage, which they attributed to the elimination of weak boundary layers and surface hydrocarbons on aluminum surfaces, resulting in a more hydrophilic surface with higher surface-free energy (SFE). Laban et al. [24] investigated the impact of various surface treatments, including degreasing, alkaline etching, O_2_ plasma, N_2_ plasma, laser, and laser+N_2_ plasma, on the fracture toughness of aluminum/fiberglass epoxy-reinforced laminates. Their findings showed that the combination of laser and N_2_ plasma yielded the highest improvement in fracture toughness, as plasma treatment generated a contamination-free surface without roughening. Lin et al. [25] confirmed that plasma treatment can remove surface contaminants, increase surface roughness and area, and form polar functional groups, strengthening the interaction between polymer and metal surfaces. Compared to other gas plasma treatments, nitrogen plasma treatment of aluminum alloy sheets yields higher strength values for Al/glass fiber/polypropylene laminates. This is attributed to the generation of C-N and C=N functional groups. Plasma treatment can be extended to GFRTP composites, in addition to its use on aluminum alloys. Dighton et al. [26] examined the effects of atmospheric pressure plasma treatment on the surface properties of GFRTP. They observed that plasma treatment had a positive impact on the surface wettability of GFRTP through the removal of surface contaminants and the increase in surface oxygen-containing chemicals. Schäfer et al. [27] investigated the effectiveness of atmospheric-pressure plasma treatment for the adhesive bonding structure of PA6 composites with polyurethane adhesives. Their study revealed that plasma treatment resulted in high-shear-strength plasma-treated joints, which was attributed to the surface enlargement caused by plasma etching and the surface functionalization with oxygen-containing groups. Zaldivar et al. [28] applied various surface treatments, including solvent wiping, abrasion, and plasma treatment, to the surface of GFRTP. Their results showed that the crack delamination resistance of plasma-treated GFRTP joints was significantly improved compared to that of the other two surface treatments. This improvement was attributed to the increase in the carboxyl content concentration on the surface of GFRTP due to plasma treatment. According to the research outlined, the enhancement in interfacial bonding strength between GFRTP and aluminum alloy after plasma treatment can be attributed to various factors, including the removal of surface contaminants, the enlargement of surface area, and the functionalization of the surface with polar groups. The effects of these parameters on surface roughness, chemical composition, and surface energy must be carefully studied to identify the optimal conditions for enhancing interfacial adhesion. Additionally, the compatibility of plasma treatment with other surface modification techniques should be investigated to develop effective surface modification strategies for FMLs.

The metal–composite interface is a critical factor in shaping the properties of FMLs, and metal surface treatment plays a substantial role in this interface. Therefore, research on the modification and characterization of metal surfaces needs to be conducted to achieve appropriate bonding at the metal–composite interface and enhance knowledge in this field. Chemical treatment of aluminum alloy surfaces can be utilized to enhance adhesion performance by increasing surface porosity and creating mechanical interlocking. However, the high viscosity and limited fluidity of the epoxy resin make it challenging for it to penetrate the porous structure of chemically treated aluminum alloy. Complete wetting of one material by another is necessary to enable effective mechanical interlocking between the two materials. This can be achieved by introducing hydrophilic functional groups via plasma treatment to increase the wettability of surfaces. Therefore, the aim of this study was to enhance the bonding strength between aluminum alloy and GFRTP by simultaneous chemical treatment and plasma surface treatment. This study focuses on the influence of discharge power, exposure time, and working gas in plasma treatment on the surface composition and morphology of aluminum alloy sheets. The bonding strength of GFRTP/aluminum alloy was subsequently examined to validate the efficacy of plasma treatment.

## 2. Experimental Procedure

### 2.1. Materials

AA 5052-H32 aluminum alloy sheets at a cold-rolled thickness of 1.5 mm were supplied by China Steel Co., Ltd. (Kaohsiung, Taiwan). Standard chemicals were purchased from Echo Chemical Co., Ltd. (Toufen, Taiwan) The 3 mm GFRTP composite was provided by InnoPeak Advanced Materials Co., Ltd (Taichung, Taiwan). The GFRTP material used for this investigation was based on 12 prepregs of plain-weave-woven E-glass/polycarbonate composites. The nominal volume fraction o reinforcing fibers was equal to about 50%.

### 2.2. Surface Treatment

In this study, aluminum alloy sheets that have not undergone any surface treatment are designated as A. Prior to etching, the aluminum alloy sheets were cleaned in a solution composed of 35 wt.% HNO_3_ and 5 wt.% NaOH at 75 °C for 2.5 min and then thoroughly rinsed in deionized water. The chemical etching process was performed using an etchant, which was a mixture of 15 wt.% FeCl_3_ and 10 wt.% H_2_SO_4_ with deionized water. After etching, the foil was rinsed in deionized water and dried in air overnight at room temperature. These chemically treated aluminum alloy sheets are designated as B. Afterward, the aluminum alloy sheets were loaded into an inductively coupled plasma chamber and exposed to argon, oxygen, and nitrogen plasma at a flow rate of 25 sccm/min. The details of the plasma parameters are listed in Table 1.

### 2.3. Structure Analysis

The surface morphology of the aluminum alloy sheets was examined using field-emission scanning electron microscopy (SEM, JEOL JSM-IT200, JEOL Ltd., Tokyo, Japan) with energy-dispersive spectroscopy. Surface roughness was measured using a surface roughness-measuring instrument (DektakXT, Bruker Corp., Billerica, MA, USA). The color of the aluminum alloy sheets was observed by optical microscopy (OM). The contact angles of water and ethylene glycol on the aluminum alloy sheets were measured using the sessile drop method by a Fat 200 contact angle system. The Owens–Wendt method was used to determine the SFE. The chemical compositions of the coatings were determined by electron spectroscopy (ESCA, PHI 5000 VersaProbe, ULVAC-PHI, Inc., Chigasaki, Japan) with monochromatic Al Ka radiation. Generally, the sample surface should be sputter-cleaned prior to ESCA measurement to remove any surface contamination, but this step can also change the surface composition. Therefore, in this case, the samples were not sputter-cleaned before ESCA measurement to ensure that the surface composition was not altered. However, the polar functional groups generated by plasma treatment inevitably underwent reduction because of the 2-day time lag between plasma treatment and ESCA analysis. Moreover, the aluminum alloy surface re-adsorbed hydrocarbon contaminations. The above factors affected the accuracy of the results obtained through ESCA analysis.

### 2.4. Lap-Shear Tensile Test

The GFRTP (40 mm × 10 mm × 3 mm) and surface-treated aluminum alloy sheet (45 mm × 18 mm × 1.5 mm) were bonded via hot-pressing technology at a bonding area of 10 mm × 5 mm. When the required bonding temperature of 190 °C was attained, the bonding force of 50 kgf/cm^2^ was applied, and the sample was cooled by compressed air under constant load. The bonding time was set to 300 s. The lap shear tensile test followed the guidelines of the standard ISO19095 using the aforementioned GFRTP and aluminum alloy sheet. A universal testing machine was used for the test. The maximum tensile force was measured, and the bonding strength was calculated. At least three tests were carried out for each parameter.

## 3. Results and Discussion

**Figure 1:**
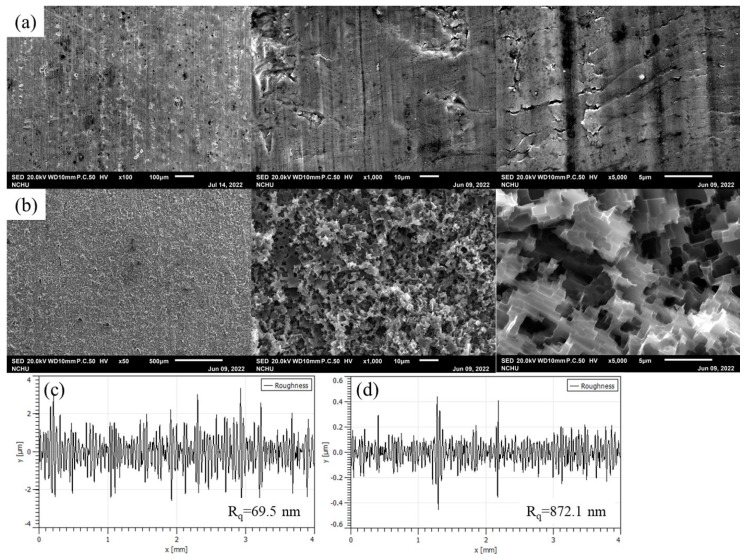
SEM images (**a**,**b**) and surface roughness (**c**,**d**) of aluminum alloy sheet: (**a**,**c**)—aluminum alloy A; (**b**,**d**)—aluminum alloy B.

**Figure 2:**
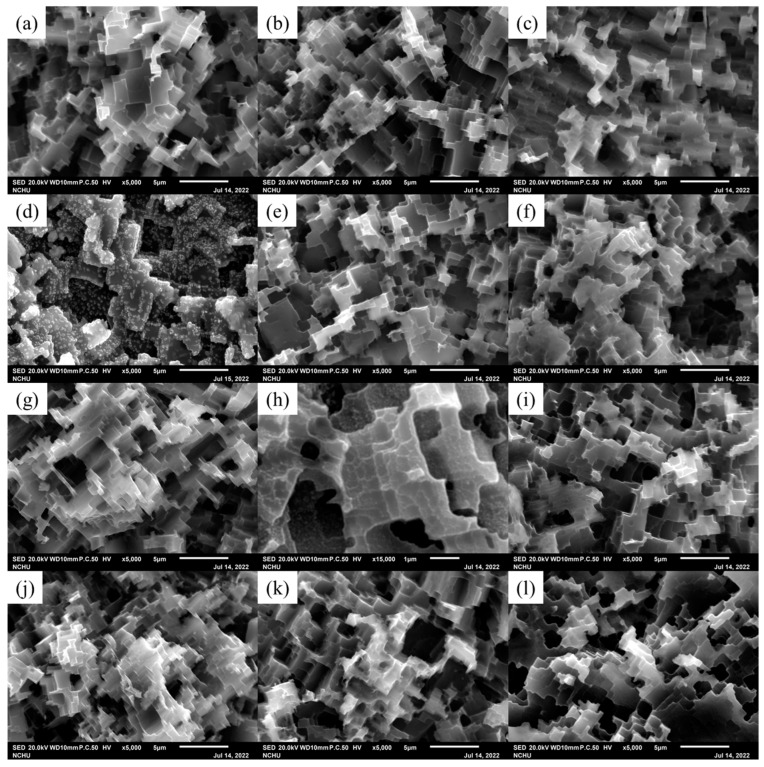
FSEM images of aluminum alloy sheets with different plasma surface treatments: (**a**) A1, (**b**) A2, (**c**) A3, (**d**) A4, (**e**) O1, (**f**) O2, (**g**) O3, (**h**) O4, (**i**) N1, (**j**) N2, (**k**) N3, and (**l**) N4.

**Figure 3:**
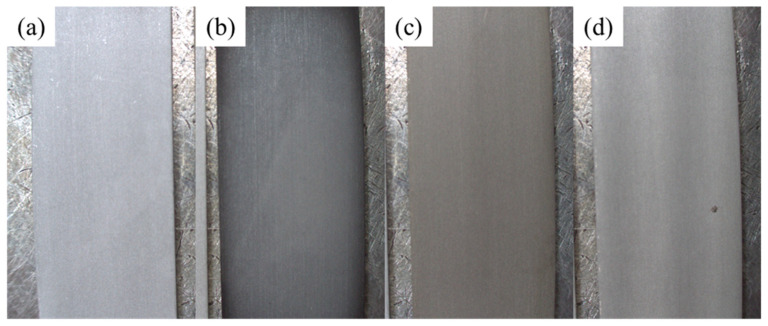
OM images of aluminum alloy sheets with different plasma surface treatments: (**a**) B, (**b**) A4, (**c**) O4, and (**d**) N4.

Figure 1 shows the SEM images and surface roughness of aluminum alloys A and B. The surface of aluminum alloy A was roughly smooth with a surface roughness of 69.5 nm and had defects, such as pores, cracks, and grooves on the surface. After the chemical etching treatment, the surface of aluminum alloy B had extensive corrosion, and the original surface defects and rolling marks completely disappeared and were replaced by a cubic pit-like corroded surface with a surface roughness of as high as 872.1 nm, which is consistent with many findings in the literature [29,30]. Figure 2 shows the SEM images of the aluminum alloy sheets treated by low-temperature plasma in different atmospheres at different discharge powers and exposure times. Regardless of the plasma treatment atmosphere, there was no significant change in the surface morphology of the aluminum alloy (for treatments A1, A2, A3, O1, O2, O3, N1, N2, and N3) after a short duration of plasma treatment, even when the discharge power was increased from 50 W to 300 W. However, when the plasma atmosphere was argon and the discharge power was 300 W, the surface morphology of the aluminum alloy changed considerably by in that the exposure time continuously increased to 300 s (A4). The surface morphology of the aluminum alloy was no longer composed of cubic pits but was replaced by an irregular glass-like morphology with scattered particles. In addition, Figure 3 displays that the color of the aluminum alloy changed from silver to a dark color. The bombardment of high-energy Ar ions for a long duration also raised the surface temperature of the aluminum alloy. These phenomena imply that the surface of the aluminum alloy had undergone remarkable changes under a long duration of high-power plasma treatment. Yi et al. [31] proposed that the surface integrity of the metal can be compromised by excessive energy or a prolonged duration of plasma treatment. When the plasma atmosphere was changed to oxygen, the surface of the aluminum alloy was only partially transformed into a glass-like morphology with scattered particles (O4). It displayed a reduced degree of color change and appeared gray. When the plasma atmosphere was changed to nitrogen, the surface of the aluminum alloy only underwent minor alterations, and most of it retained its silver color (N4). The results imply that different plasma treatment atmospheres have different energy transfer effects on the surface of aluminum alloy, and the order is argon > oxygen > nitrogen. This contrast may be explained by the difference in the bond dissociation energies of the atmosphere. The bond dissociation energies of gaseous argon, oxygen, and nitrogen are 0, 498, and 945 kJ/mol, respectively [32]. The bond dissociation energy of nitrogen molecules is very large, which can lead to large energy consumption for plasma generation, reducing the flux of ionized particles and their average kinetic energy upon striking the surface of aluminum alloy. Yi et al. [31] also found that nitrogen plasma treatment causes less damage to the surface of the sample when compared to argon plasma treatment.

**Figure 4:**
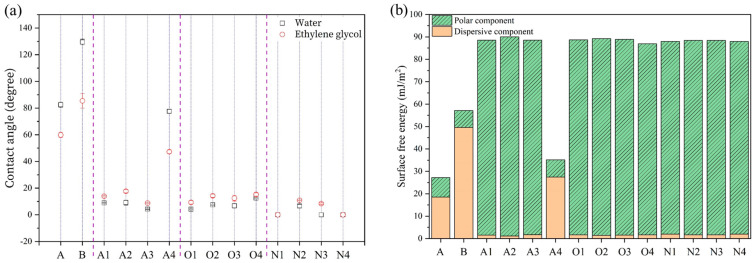
(**a**) Contact angles of water and ethylene glycol and (**b**) SFE of aluminum alloy sheets with different plasma surface treatments.

The wettability between metal and composite materials is an important factor in adjusting their interface adhesion strength. Figure 4a shows the contact angles of water and ethylene glycol on the aluminum alloys treated by different plasma treatments. The contact angle of aluminum alloy A was 82°, indicating a hydrophobic surface. The low surface wettability may be attributed to the hydrocarbon contamination on the surface. The water contact angle of aluminum alloy B was 130°. The cubic pit-like corroded surface drastically increased the contact area, which formed a more hydrophobic surface. According to the Wenzel model, in the case of hydrophobic surfaces, the greater the surface roughness, the poorer the surface wettability [33]. When aluminum alloy B was treated with plasma, the water contact angle decreased greatly, and the surface became very hydrophilic. In the case of argon plasma treatments, the main plasma treatment process involves sputtering by incident argon species, so it can remove hydrocarbon contaminations from the surface of aluminum alloys [23]. However, it may cause a large number of dangling bonds during plasma treatment [34]. Once returned to the atmosphere, the dangling bonds immediately react with oxygen or moisture in the air to form a hydrophilic surface with oxygen-rich and hydroxyl-rich surface functional groups. As a result, the water contact angle of aluminum alloys Al, A2, and A3 were lowered to 9°, 9°, and 4°, respectively. However, when the discharge power was 300 W and the exposure time was up to 300 s, the degree of surface wettability improvement decreased. The water contact angle of aluminum alloy A4 was 77°. The reason for the sudden decrease in surface wettability will be discussed later. In the case of oxygen plasma treatments, the oxygen radicals generated in the plasma produce CO, CO_2_, and H_2_O by reacting with carbon compounds, resulting in a more efficient removal of hydrocarbons from the surface of the aluminum alloy [35,36]. As a result, the water contact angle of aluminum alloys Ol, O2, O3, and O4 were lowered to 4°, 7°, 6°, and 13°, respectively. The high power and long exposure time of the oxygen plasma treatments also reduced the improvement in surface wettability, but their impact was much less than that of the argon plasma treatment. In the case of nitrogen plasma treatments, UV lines at shorter wavelengths generated from the decay of a range of neutral nitrogen metastable species trigger the disassociation and desorption of hydrocarbons, accelerating the plasma cleaning activity [37,38]. As a result, the water contact angle of aluminum alloys Nl, N2, N3, and N4 decreased to 0°, 7°, 0°, and 0°, respectively. The contact angle of ethylene glycol after plasma treatment exhibited a similar trend to the water contact angle. The great increase after plasma exposure was observed in the polar component. From the above data, the nitrogen plasma treatment was more effective in improving surface wettability than the argon and oxygen plasma treatments were [25,39,40].

The SFE values of the aluminum alloys treated by plasma treatments were remarkably increased compared with aluminum alloy B. Figure 4b shows the SFE values of the aluminum alloys treated by different plasma treatments. The SFE of the plasma-treated aluminum alloys had a similar value of about 87–90 mJ/m^2^, which was 3.1–3.4 times higher than that of aluminum alloy B. The polar and dispersive components of the plasma-treated alloys displayed a large increase, triggering more complete surface wettability. However, although plasma treatment can increase surface wettability, this effect is time-dependent and can diminish over time. Plasma-generated polar functional groups tend to reorient toward the bulk of the film or move toward the material matrix through diffusion [41]. Furthermore, hydrocarbon contamination in the air can re-adsorb onto the surface of aluminum alloys, resulting in reduced surface wettability [42].

**Figure 5:**
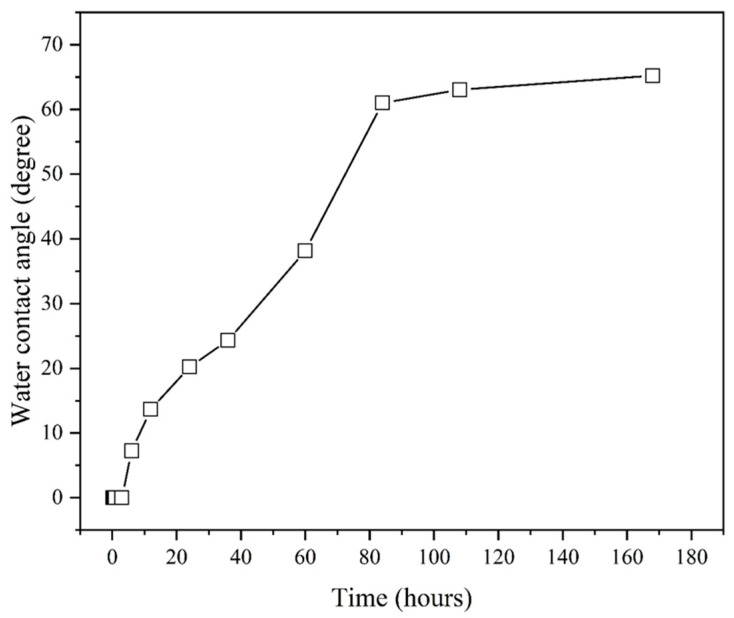
Water contact angle of aluminum alloy sheet as a function of exposure time for nitrogen plasma.

Figure 5 shows the water contact angles of the aluminum alloys treated by nitrogen plasma treatments as a function of exposure time. After nitrogen plasma treatment, the water contact angle was stable at 0° for 3 h. The water contact angle then increased rapidly to 61° with the exposure time up to 84 h, after which it tended to increase steadily. This finding indicates that plasma-treated aluminum alloys must be combined with GFRP within 3 h to obtain a better bonding strength.

**Figure 6:**
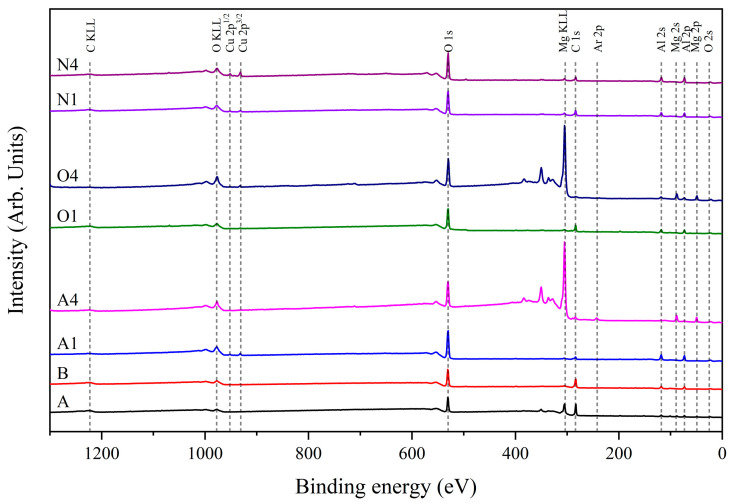
ESCA wide-scan spectra of aluminum alloy sheets treated with different plasma treatments.

ESCA analysis was adopted for the primary evaluation of the influence of plasma treatment on the chemical composition of the surface of the aluminum alloy sheets. Figure 6 shows the ESCA wide-scan spectra of the aluminum alloy sheets treated by different plasma treatments. Their chemical composition was listed in Table 2. The following phenomena were observed: (i) Aluminum alloy A had a lower Mg content but a higher Al content than aluminum alloy B did, which means that the element Mg is preferentially corroded during the chemical etching process. The corrosion mechanism of active ions is based on galvanic corrosion. From the standard electrode potentials of metals in Table 3 [43], the potential difference between the ions of Mg and other active ions is large, resulting in preferential corrosion. Moreover, Al and Mg are two metals that can experience galvanic corrosion when used together [44]. When Mg with lower oxidation–reduction potential comes into contact with Al with higher oxidation–reduction potential, the Al will become the cathode and be protected, while the Mg will become the anode and be corroded. The corrosion rate of the Mg metal will be higher than if the two metals were not in contact. (ii) The C 1s signal of the plasma-treated aluminum alloys declined dramatically, indicating that plasma treatment can effectively remove the hydrocarbon contamination on the surface. (iii) As mentioned above, the surface morphology and color of aluminum alloy changed remarkably under a long duration of high-power plasma treatment, which can be ascribed to the substantial modification in the surface chemical composition. Al content was greatly reduced whereas Mg content was relatively increased in aluminum alloy A4, which means that the element Al is preferentially etched by plasma. However, the actual plasma etching rate of Al was lower than that of Mg. A study that calculated sputtering yields for target materials made of aluminum and magnesium revealed that the theoretical yield for magnesium is approximately twice that of aluminum [45]. Duchoslav et al. considered that plasma treatment generates acidic species that can react with MgO surfaces exhibiting poor acid resistance. The reaction leads to the formation of highly soluble mixtures, such as MgOH, which ultimately causes the loss of Mg from the surface [46]. To date, the published literature provides limited information regarding the significant loss of aluminum during plasma treatment. It is speculated that the plasma treatment at high power over a long time caused the surface temperature of the aluminum alloy to rise sharply, resulting in Al diffusing outward and being preferentially etched. (vi) The element Ar could be detected on the surface of aluminum alloy A4. This phenomenon confirms that the argon plasma treatment under high power and a long duration had a very strong bombardment effect on the surface of the aluminum alloy, leading to the trapping of Ar ions in the near-surface region of the aluminum alloy. These trapped Ar ions reduced the surface area of the aluminum alloy in contact with water and hindered the formation of polar functional groups, thus decreasing the alloy’s hydrophilicity. The former can be supported by the framework of the Cassie–Baxter impregnating state [47]. The latter can be related to the type of surface defects. Saini et al. suggested that Ar^+^ ion irradiation can induce the formation of oxygen vacancies on TiO_x_ surfaces and make this system become hydrophobic [48].

**Figure 7:**
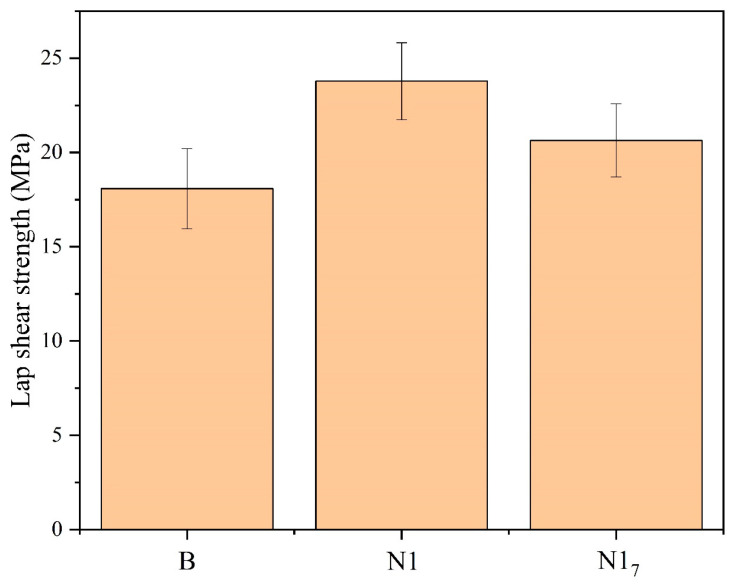
Bonding strength of GFRTP/aluminum alloy bonding with different plasma treatments.

Figure 7 shows the bonding strength of GFRTP/aluminum alloy bonding with different plasma treatments. The bonding strength of uncorroded aluminum alloy A was almost zero. After the chemical etching treatment, the surface roughness of aluminum alloy B increased, which greatly improved the bonding strength to up to 18.1 MPa. Higher surface roughness in aluminum alloys can result in stronger adhesion between the two materials. This greater bonding is attributed to the increased number of contact points and the mechanical interlocking or “hooking” effect created by the rough surface, which can provide a larger interfacial area and greater resistance to interfacial separation. Many theoretical and experimental studies demonstrated the positive correlation between surface roughness and bonding strength [6,7,25,49]. However, the relationship between surface roughness and adhesion strength is not always straightforward and also depends on the chemical nature of the metal surface. After the nitrogen plasma treatment, the bonding strength of the GFRTP/aluminum alloy increased to 23.8 Mpa. The bonding strength of aluminum alloys and GFRTP is typically associated with the contact angle (SFE). During the bonding process between the aluminum alloy and GFRTP, the contact angle can affect the diffusivity of the epoxy resin and thus affect the bonding strength. When the contact angle is smaller, the liquid covers a greater area on the surface of aluminum alloys, making it easier for the epoxy resin to diffuse into the tiny recesses on the surface and thus form a stronger bond. In this study, a stronger bonding strength was achieved through the synergistic effect of mechanical bonding facilitated by the surface roughness and chemical bonding via functional groups, which existed between the aluminum alloy sheet and GFRTP. However, when the nitrogen plasma-treated aluminum alloy was left for 7 days before being hot-pressed with GFRTP, the bonding strength decreased to 20.6 MPa. The decrease in SFE impeded the effect of mechanical interlocking greatly on the state of incomplete wetting, resulting in the decrease in bonding strength. This finding implies that timeliness in plasma treatment is very important for ensuring the effectiveness of processing.

## 4. Conclusions

The study investigated the effects of different plasma treatments on the surface morphology, wettability, and bonding strength of aluminum alloys. Plasma treatment considerably improved the surface wettability of the aluminum alloys, but excessive plasma treatment reduced the improvement in the hydrophilicity of the aluminum alloys. Among the various plasma treatments, the nitrogen plasma treatment was the most effective at improving surface wettability, whereas the argon plasma treatment was utilized for changing surface topography. The improved wettability was time-dependent and could diminish over time due to the re-adsorption of hydrocarbon contamination in the air. The bonding strength of GFRTP/aluminum alloy was strongly influenced by the surface wettability, and the bonding strength decreased over time after plasma treatment. These findings suggest that plasma treatment can be a useful method for improving the surface properties and bonding strength of aluminum alloys, but timely processing is important to ensure the effectiveness of processing.

## Figures and Tables

**Table 1:** Plasma treatment parameters for aluminum alloy surface.

Sample	Plasma Gas	Discharge Power (W)	Exposure Time (s)
A1	Ar	50	30
A2		300	30
A3		300	120
A4		300	300
O1	O_2_	50	30
O2		300	30
O3		300	120
O4		300	300
N1	N_2_	50	30
N2		300	30
N3		300	120
N4		300	300

**Table 2:** The surface composition of all samples.

	A	B	A1	A4	O1	O4	N1	N4
[Al]/([Al]+[Mg])	57.3	96.4	97.6	9.1	95.4	24.7	96.8	97.0
[Mg]/([Al]+[Mg])	42.7	3.6	2.4	90.9	4.6	75.3	3.2	3.0
[C]/([C]+[O])	61.8	53.3	23.7	21.8	40.1	27.7	32.4	25.6

**Table 3:** Oxidation-reduction potentials of selected metallic elements [43].

Element	Reaction	Oxidation-Reduction Potential (V)
Magnesium	Mg^2+^ + 2e^−^ → Mg	−2.37
Aluminum	Al^3+^ + 3e^−^ → Al	−1.66
Manganese	Mn^2+^ + 2e^−^ → Mn	−1.18
Iron	Fe^2+^ + 2e^−^→ Fe	−0.44
Copper	Cu^2+^ + 2e^−^ → Cu	+0.34

## Data Availability

Data are contained within this article.
